# The abundances of carbon and nitrogen in the atmospheres of classical Be stars

**DOI:** 10.1007/s10509-026-04538-8

**Published:** 2026-01-26

**Authors:** Geraldine J. Peters, Kenneth G. Gayley, Rina G. Rast, Jorick S. Vink, Jeremy J. Drake

**Affiliations:** 1 Department of Physics and Astronomy, University of Southern California, University Park Campus, Los Angeles, 90089-0484 CA USA; 2 Department of Physics and Astronomy, University of Iowa, Iowa City, 52242 IA USA; 3 Physics and Astronomy, The University of Western Ontario, 1151 Richmond Street, London, N6A 3K7 Ontario Canada; 4 Armagh Observatory and Planetarium, College Hill, Armagh, BT61 9DG Northern Ireland UK; 5 Lockheed Martin Solar and Astrophysics Laboratory, Advanced Technology Center, 3251 Hanover Street, Palo Alto, CA 94304 USA

**Keywords:** Algol binaries, Close binary stars, Be stars, OB subdwarf stars, Circumstellar matter, Early-type emission line stars, Stellar mass loss, UV astronomy

## Abstract

Hot stars born as rapid rotators are expected to induce meridional currents that mix hydrogen from the envelope into the core and return CNO-cycle processed material to the envelope, which should enhance the N at the surface at the expense of C and possibly also O depending on the ambient conditions. But the photospheric C and N abundances could also be influenced by mass transfer in a close binary system which spins up the mass gainer and deposits either processed or unprocessed material to its surface depending on just how much material has been peeled off the mass donor. We focus on the chemical composition of Be star photospheres to infer the present and past evolution of rapidly rotating early B stars. To mitigate the effects of gravity darkening and photospheric line blending on the abundances, we chose 8 Be stars with low $v\sin i$ that have good high-resolution FUV spectra in the *IUE* archive. We carried out a conventional NLTE abundance analysis of selected N iii, N i, and C iii lines in the FUV spectral region. We find clear evidence that the C iii 1176 Å multiplet is weak in the core region in most program stars, suggesting CNO processing. However, in all cases we infer a N abundance that is solar or less, raising a conundrum as to what happened to the “missing C.” Since a similar pattern of weak C yet normal N is also found in the mass gainer in some Algol binaries, there appears to be an emerging challenge to explain this apparent abundance anomaly. We speculate that the excess N from CNO processing might be converted into O (and perhaps on to Ne) by fusion with He in the hot but low-density regions either in the trail of ashes just outside the receding carbon-fusing core, or in He-shell flash regions, of a highly evolved mass loser in its final stage of mass transfer.

## Introduction

The recent discovery and confirmation that about 30 well-known bright Be stars have an sdO companion (Wang et al. 2021, 2023) has reinforced earlier discussion in the hot star community that many Be stars are interacting binaries spun up to fast rotation by mass transfer. The sdO object would then be interpreted as the core of the mass donor, with most of its envelope material stripped during mass exchange. Furthermore, it is reasonable to suspect that the Be + sdO systems might have been massive^1^ Algol binaries in their earlier life.

If massive Algols become Be + sdO systems, the atmospheres of the mass gainers of both types of binaries should also show non-solar abundances of C and N in the post-mass-reversal epoch because the mass loser can transfer gas that has been mixed through CNO processing regions in the evolving mass donor. This should produce C depletion, and concomitant N enrichment if the CNO processed gas experiences no further fusion stages. In this paper, we use archival FUV spectra to assess limits on the C depletion, and any corresponding N abundance changes, in the atmospheres of classical Be stars. Our goal is to test the hypothesis that these systems show a C and N abundance pattern consistent with having an Algol past, and to constrain the fusion mixing that occurred in that past. We also characterize the future observational improvements needed to better solidify these conclusions.

The structure of the paper is to present our analysis strategy in Sect. 2, and in Sect. 3 give details concerning our correction of the observed FUV flux for continuous emission from the disk. The modeling technique, including the determination of the stellar input parameters and errors, is presented in Sect. 4. The individual abundance results and information on each program star’s disk activity and prominence during the *IUE* era (when the observations were made) is presented in Sect. 5. The N and C abundances determined or inferred for the photospheres and circumstellar material of early B-type Algol primaries is discussed in Sect. 6. Comments concerning whether N enhancement and C depletion at the photosphere of the star are a signature of single or close binary evolution appear in Sect. 7. The paper includes a discussion of how future UV spectroscopic capabilities can clarify our understanding of stellar evolution on the upper main-sequence. Some final thoughts, and a speculation of how C can be reduced without showing up as an N enrichment, are presented in Sect. 9.

## Project strategy

Interior models for the structure and evolution of rapidly rotating OB stars predict a photospheric enrichment of N and a deficiency of C as a result of the mixing of CNO-processed material from the star’s core region with the original surface material (Maeder and Meynet 2000).^2^ For pursuing an abundance study to test this prediction, we chose the FUV spectral region, as it provides several strong lines from the higher ionization species. Also, the FUV flux is bright in OB stars, barring excessive interstellar and/or circumstellar reddening.

That said, analyses of Be star spectra using standard spectrum synthesis techniques have some challenges that are not encountered in abundance studies of sharp-lined, non-emission B stars. Some of these include the treatment of blended, rotationally-broadened lines, determination of the microturbulence parameter, correction for line and continuum emission from the disk and possible shell absorption, and latitudinal variation of $\mathrm{T_{eff}}$ and log $g$. Also, if the Be star has an sdO companion, its FUV flux must be subtracted from the observed spectrum to correctly characterize the line depths.

Here we present results from a spectrum synthesis study of high resolution FUV spectra of Be stars secured from the *IUE* archive^3^ The Hubeny/Lanz NLTE codes TLUSTY/SYNSPEC, Hubeny (1988) and Hubeny and Lanz (1995), and the Lanz/Hubeny model atmospheres for B stars (Lanz and Hubeny 2007) were employed. The FUV offers an advantage over the optical region, since there is less influence from continuous disk emission, and some FUV lines (e.g., N ii-iii and C ii-iii) are intrinsically stronger.^4^

If the study is focused on the so-called *pole-on* Be stars, we can mitigate the effects of line blending due to rotational broadening, while still being confident that we are dealing with rapid rotators. Even so, shell or interstellar lines can sometimes significantly blend with the lines that are the focal point of the study and make it difficult to place the continuum. For this study, we have focused on Be stars with values of $v\sin i$ $\mathrm{<~160~km~s^{-1}}$ in which the integrated flux is dominated by FUV light and the effect of latitudinal parameter variation is minimized (Frémat et al. 2005).

We selected *IUE* spectra taken in the short-wavelength, high-resolution mode (SWP HIRES, $\lambda /\Delta \lambda =10{,}000$). The IUEDAC software^5^ was used to prepare the observed profiles that would be compared with the model predictions. Each SWP HIRES spectrum was inspected for quality and the best ones were coadded to improve the S/N. Generally we did not include observations that were carried out with the *IUE* small aperture unless all the usable spectra were taken in the latter mode. The coadded spectrum was then continuum normalized. Continuum light from the nearly *face-on* disk was subtracted from the coadded spectrum as was the flux from an expected sdO companion. A typical value for the percentage of FUV disk flux relative to the Be star is 2-3% (cf. Sect. 3) and an sdO companion would contribute another 5% (Wang et al. 2021). For this study we subtracted 8% of the light from each spectral region analyzed^6^ to approximate the disk and sdO star backgrounds.

The Be stars considered in this study are in the B1-B3 range and include 11 Cam (B3 Ve), FW CMa (B3 Ve), 16 Peg (B3 Ve), $\omega $ CMa (B2.5 Ve), 31 Peg (B2 IVe), $\mu $ Cen (B2 IV-Ve), MX Pup (B1.5 IVe), and $\chi $ Oph (B1.5 Ve). Additional information can be found in Table 1.

**Table 1:** The Program Stars

Star	HD	Spectral type	*v* sin *i* $\mathrm{km~s^{-1}}$	$\mathrm{T_{eff}}$ K	log *g* cgs	Number coadded
11 Cam	32343	B3 Ve	95	16,000	4.0	3
FW CMa	58343	B3 Ve	40	16,000	4.2	4
16 Peg	208057	B3 Ve	100	17,000	4.0	4
*ω* CMa	56139	B2.5 Ve	85	19,000	4.0	2
31 Peg	212076	B2 IVe	100	20,000	3.5	3
*μ* Cen	120324	B2 IV-Ve	155	21,000	3.9	26
MX Pup	68980	B1.5 IVe	130	25,000	3.9	3
*χ* Oph	148184	B1.5 Ve	150	27,000	3.9	2

## The flux contributed by the disk

Be star disks can add to the brightness of the system through free-free and bound-free emission (Gehrz et al. 1974). This effect depends on density, inclination, and wavelength; denser disks viewed near face-on will be the brightest, and emission at longer wavelengths is produced across regions that extend further into the disk (Carciofi and Bjorkman 2006; Haubois et al. 2012; Vieira et al. 2015). Emission at UV wavelengths is therefore produced very near the stellar surface, and could be significant for objects viewed at low inclinations. In order to properly account for the disk’s contribution to the continuum flux at the wavelengths relevant to this work, we used models created by the 3D Monte Carlo radiative transfer code, hdust (Carciofi and Bjorkman 2006, 2008). We modeled a B2 star with the stellar parameters shown in Table 2, consistent with the values in Cox (2000), and a disk radius of 25 equatorial radii of the Be star. We tested different stellar rotation rates $W$, defined by $W = v_{\mathrm{{rot}}}/v_{\mathrm{{orb}}}$, where $v_{\mathrm{{rot}}}$ is the rotation rate of the star and $v_{\mathrm{{orb}}}=(GM/R)^{1/2}$ is the Keplerian velocity at the stellar equator. To provide a range of possible values expected for disks around B2 stars, we considered models with disks of high and moderate densities, as estimated from Fig. 8(a) of Vieira et al. (2017). Here, $\rho _{0}$ refers to the midplane density of the disk at the stellar equator.

**Table 2:** Stellar and disk parameters used in the hdust models

Parameter	Value
*M* (M_⊙_)	9.11
$R_{\mathrm{p}}$ (R_⊙_)	5.33
$T_{\mathrm{eff}}$ (K)	21,000
*L* (L_⊙_)	4950
$\rho _{0}$ (10^−11^ g cm^−3^)	0.1, 1

**Fig. 1:**
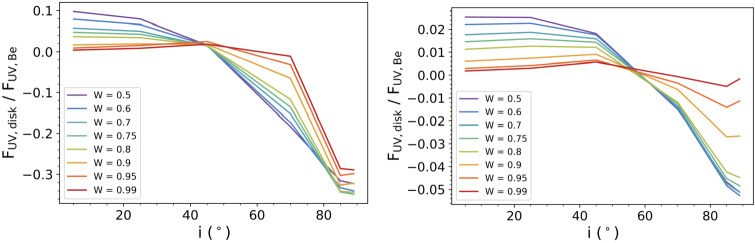
Ratio of the UV (1170-1300 Å) flux contributed by the disk to that of a diskless Be star of spectral type B2. We show the results for a high density disk in the left panel and a moderate density disk in the right panel. Different stellar rotation rates $W$ are shown, as indicated in the legend

Figure 1 shows the ratio of the UV flux, computed as the mean across 1170-1300 Å, produced by the disk to the UV flux produced by the Be star over a range of inclinations, from near pole-on to near edge-on. This ratio was found for different stellar rotation rates, as indicated in the legend. When viewed at the smallest tested inclinations, the disk can contribute nearly 10% of the stellar flux at low rotation rates for a high density disk, or more than 2% for a disk of moderate density. Stars rotating near the critical limit emit more flux from the poles and the disk’s contribution is much smaller, so the disks surrounding the fastest-rotating Be stars contribute much less to the continuum. As the inclination angle at which the system is viewed increases, the disk contributes less to the system brightness and eventually begins absorbing rather than emitting light. Since we focus on low inclinations, we expect the disk to add light, but not more than a few percent according to Fig. 1.

## The modeling

The values for $\mathrm{T_{eff}}$, log $g$, and $v\sin i$ of each star (corrected for gravity darkening and geometrical flattening) are given in Frémat et al. (2005). We used these parameters for our initial calculations and then adjusted them based on the observed strengths of the Si ii, Si iii and Si iv features. For the cooler Be stars log $g$ was estimated from the outer red wing of Ly$\alpha $. The $v\sin i$ was determined from several moderately-strong, isolated lines. The most uncertain parameter is the microturbulence. It has been known for some time now that the value of the microturbulence determined from the UV lines in early B stars is usually smaller than that suggested by their optical O II features. This probably means that the deeper atmospheric layers in these stars are more quiescent. The effect is illustrated in Peters and Meynet (2011) where the value of $\mathrm{V_{turb}}$ from the UV studies of Proffitt and Quigley (2001) is plotted against the optical results for the same star Gies and Lambert (1992). Implicit is an apparent trend that $\mathrm{V_{turb}}$ increases with $\mathrm{T_{eff}}$. Based upon this plot, we adopted a $\mathrm{V_{turb}}$ of 4 $\mathrm{km~s^{-1}}$ for stars earlier than B2 and 1-2 $\mathrm{km~s^{-1}}$ for the cooler objects.

Uncertainties in $\mathrm{T_{eff}}$, log $g$, and $v\sin i$ produce errors in the derived abundances of 0.2-0.3 dex. The errors produced from the uncertainty in the microturbulence are comparable. The moderate signal-to-noise ratio of the *IUE* spectra can produce an uncertainty up to 0.5 dex for some stars with $v\sin i$ > 120 $\mathrm{km~s^{-1}}$. Large systematic errors (up to 0.5 dex) can result from the uncertainty in the placement of the continuum. This is especially true for Be stars that display extensive shell absorption. The background is also a problem at the shortest wavelengths in the *IUE* images where the echelle orders are closely spaced. However, the presence of Ly$\alpha $ at 1216 Å, which has a zero intensity at its core mostly due to interstellar extinction, gives one confidence that the background has been correctly subtracted. Of course, if the star does not have an sdO companion, there would be a systematic error in the observed profiles toward larger flux.

**Fig. 2:**
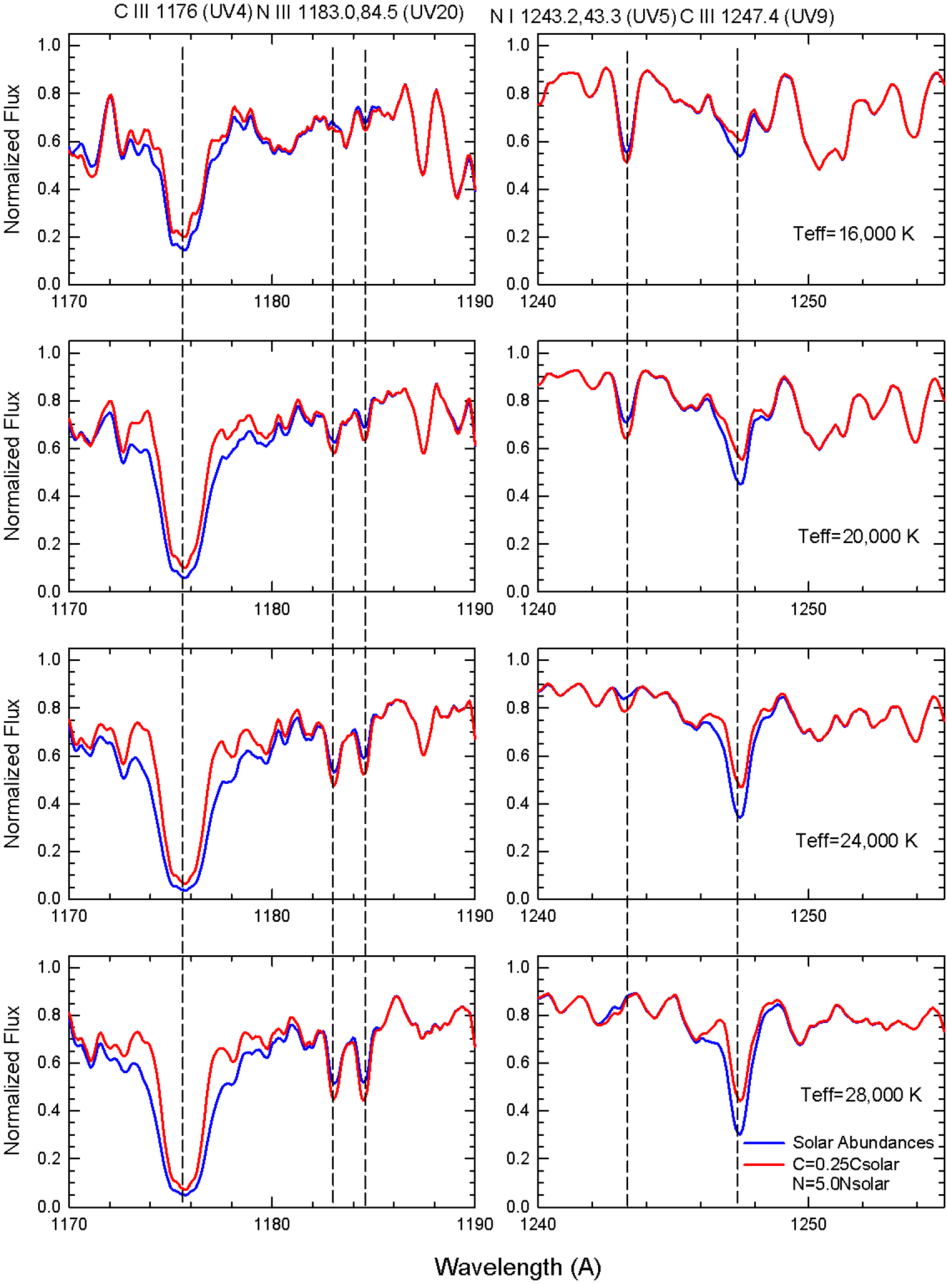
Model spectra that span a $\mathrm{T_{eff}}$ range of 16-28 kK illustrate what one would expect to observe. The calculations were carried through using a log $g$ of 3.8 (cgs units), $v\sin i$ = 100 $\mathrm{km~s^{-1}}$, and $\mathrm{V_{turb}}$ = 4.0 $\mathrm{km~s^{-1}}$

In this paper, we examine two spectral regions in the FUV that contain moderately strong to strong lines of C iii, N i, and N iii. Included are C iii 1176 Å (UV 4), N iii 1183.0 and 1184.5 Å (UV 20), and C iii 1247.4 Å (UV 9), which are prominent in the stars earlier than B2, and N i 1243.2 Å (UV 5) observed in the B2–B3 stars. In Fig. 2 model spectra spanning the temperature range of the programs stars illustrate what one might expect from the spectral fits. The model spectra computed with TLUSTY/SYNSPEC for solar abundances are overplotted with those for which the abundance of C is 25% of the solar value^7^and N is five times solar. The latter represents an estimate of an extreme condition that one might encounter if mixing from the star’s interior and/or CNO-processed material from the mass loser dominates in the mass gainer’s photosphere.

Spectral features of interest in the FUV are often blended with weaker lines from abundant ions. We estimated this excess absorption with the aid of the high-resolution ($\Delta \lambda =0.05$ Å) FUV spectra that were secured with the *Copernicus* spacecraft in the 1970s.^8^ When interpreting the line profile of the C iii 1176 Å multiplet, it is important to focus on the core of the line between 1174.5 and 1176.5 Å because just to the blue and red of this region are moderately-strong features of Si iii and Fe iii that increase in strength from B3 IV-V to B1 IV-V.

## Individual results

**Fig. 3:**
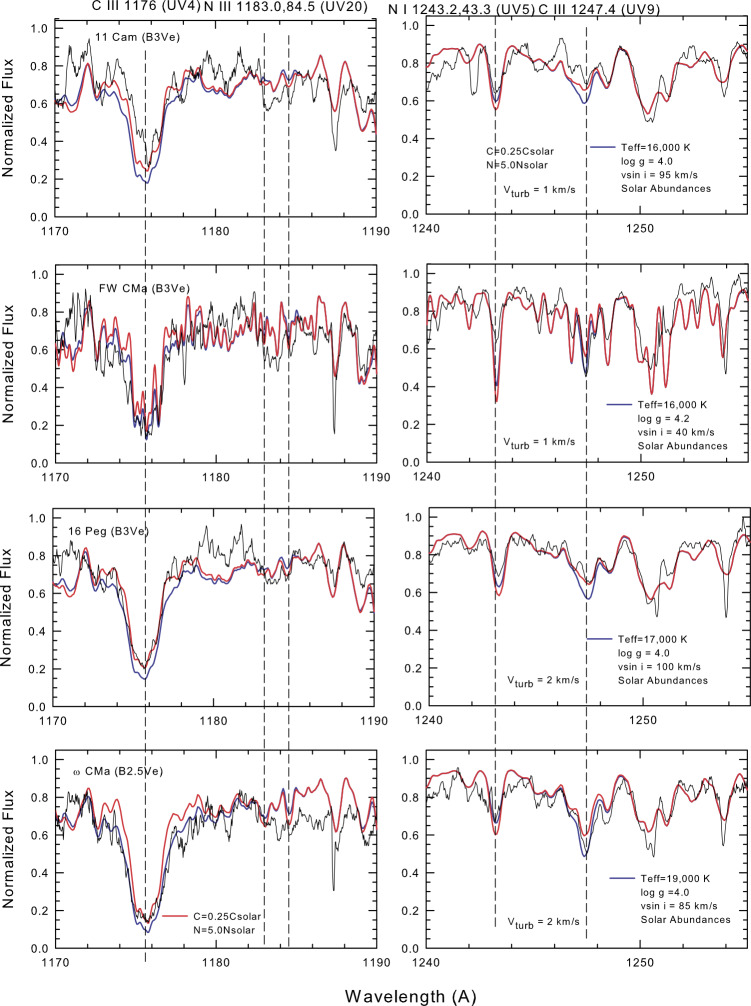
Comparison between the observed FUV line profiles for the cooler Be stars and model spectra computed with the stellar parameters indicated on the plot

**Fig. 4:**
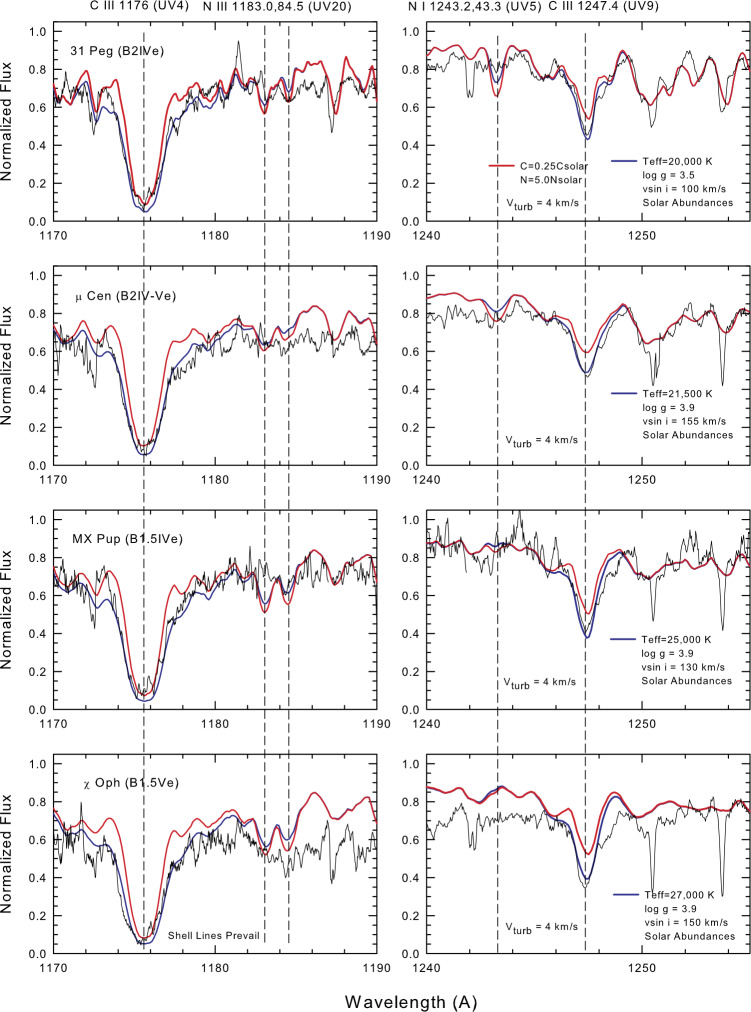
Model fits to selected observed line profiles for the hotter Be stars

Comparisons between the model spectra for each program star and the observed profiles (corrected for continuous disk emission and the presence of a typical sdO secondary) are shown in Figs. 3 and 4. The stellar parameters used to generate the model profiles are indicated on the individual plots.

**11 Cam** is the coolest Be star that we studied. The observed spectrum is a coadition of three SWP HIRES images (SWP 05927, SWP 05960, and SWP 06932). It is apparent that both C iii lines indicate a carbon abundance less than or equal to that of a CNO-processed photosphere and significantly lower than that of a photosphere with solar composition. Conditions in the photosphere are cool enough to form the N I line and considering a typical error of about 0.3–0.4 dex a near solar N abundance is indicated. One would not expect to see N iii in the star’s atmosphere.

**FW CMa** has the sharpest lines among our program stars. With a $v\sin i$ of only 40 $\mathrm{km~s^{-1}}$, its spectrum shows as much detail as many sharp-lined non-emission B stars that have historically been used to determine B star abundances. Kodaira and Scholz (1970) performed an abundance of three B3 stars, including the ultrasharp-lined star $\iota $ Her, the non-emission moderate rotator $\eta $ Hya, and FW CMa using non-line blanketed, LTE model atmospheres of the mid-1960s. They concluded (from four C ii optical lines) that carbon is underabundant by about 0.8 dex relative to the sun, 0.5 dex compared with $\iota $ Her, and 0.1 dex relative to $\eta $ Hya. They also found, from two optical lines of N ii, that nitrogen was also underabundant by about 0.4 dex relative to the solar value. Four *IUE* SWP HIRES images (SWP 06992, SWP 08605, SWP 15978, and SWP 21914) were coadded to generate the observed FUV spectrum. The fit to C iii 1176 Å was inconclusive, but C iii 1247 Å suggested a solar abundance. The continuum normalization was more uncertain for this star than for the other program stars. N i 1243 Å suggested an underabundance of nitrogen. The obvious absorption around the N iii 1183-1184.5 Å features does not originate from N iii but rather from other lines [e.g. Mn iii (UV 4 & 7)] seen in B3 atmospheres.

**16 Peg** was devoid of disk emission during the 1970-80s when the *IUE* observations were made (Slettebak 1982), so the observed spectrum was corrected only for the possible presence of an sdO object. This star thus provides us with an opportunity to assess whether the extraneous absorption that often occurs around our lines of interest originates in the photosphere or a disk. SWP 31187, SWP 32189, SWP 31518, and SWP 33664 were coadded to generate the observed line profiles. Our fits of the two C iii lines clearly indicate carbon depletion. A clean fit of N i 1243 Å is consistent with a solar abundance for nitrogen.

$\boldsymbol{\omega} $ **CMa** is a 4th magnitude Be star with a rich archive of published papers. Its complex nonradial pulsations have been modeled by Maintz et al. (2003). During the *IUE* era, Baade (1982) found a fairly stable radial velocity variation with a period of 1.365 days and concluded it was caused by nonradial pulsations. Two *IUE* SWP HIRES images (SWP 15028 and SWP 15980) were coadded to generate the observed spectrum. The core intensity of the C iii 1176 Å multiplet suggests that carbon abundance is only 25% of the solar value in the photosphere. The wings of this feature show a significant blending with Si iii (UV 30) and many weaker lines of Mn iii found in atmospheres with temperatures ranging from 18-23 kK (cf. Sect. 4). However, C iii 1247 Å indicates only a slight carbon depletion. The wings of the latter feature are somewhat deeper due to blending with weaker Si ii features. N i 1243 Å shows a solar abundance for nitrogen.

**31 Peg** was in a recovery stage from a weak emission phase when the *IUE* observations presented here were secured (Slettebak 1982). The observed *IUE* spectrum is a coadd of SWP 03828, SWP 05910, and SWP05931. The core region of the C iii 1176 Å multiplet fits well with the model computed for a reduced carbon abundance, but the C iii 1247 Å feature was well fitted with a solar abundance. The nitrogen abundance is inconclusive but the observations point more to a solar abundance.

$\boldsymbol{\mu} $ **Cen** was observed 35 times with the *IUE* SWP HIRES camera and large aperture between 1979-85. During the latter time interval the star was in the process of reestablishing its disk after a brief no-disk period in 1977. The H$\alpha $ emission strength was about five times the local continuum value in 1973 (Peters 1979). The spectral variability in $\mu $ Cen prior to 1981 is discussed in Peters (1985). In 1985 a burst of activity was serendipitously observed in the H$\alpha $ region revealing a global mass loss event on the time scale of 10 minutes (Peters 1986). Twenty-six *IUE* images were coadded to form the observed spectrum. The intensity of the core of C iii 1176 Å suggests a carbon abundance of about half of the solar value, but C iii 1247 Å is fit well with a solar abundance. The N iii and N i results are inconclusive. Considering the observational errors and different model atmospheres that were employed, the abundance results in the current investigation are in agreement with those determined by Peters (1979) from *Copernicus* FUV spectra and ground-based spectrograms taken at Lick Observatory. Peters found solar abundances for the carbon lines analyzed in this study, but C ii 1335 Å and C iii 1247 Å indicated a carbon abundance that is about ten times smaller. The N abundance appeared solar. Considering all the above spectra, there is some evidence for an underabundance of carbon in the photosphere of $\mu $ Cen.

**MX Pup** (r Pup, HR 3237) was identified as a likely binary star in the 1990s (Mennickent et al. 1994, 1997), which was confirmed by Carrier et al. (2002). However, the nature of this system remains uncertain. Its orbital period is only 5.1526 d, which is much shorter than that of a typical Be + sdO system (Wang et al. 2023) and more like that of an Algol binary. The system is likely a massive binary with a large mass ratio and very close separation in the early stages of mass transfer (Bao et al. 2025). The observed spectrum presented here is a coaddition of three SWP HIRES images (SWP 22119, 33314, and 36214). The C iii 1176 Å line suggests an underabundance of carbon, but the C iii 1247 Å feature indicates a solar abundance. One component of the N iii multiplet fits well with a solar nitrogen abundance.

$\boldsymbol{\chi} $ **Oph** displayed strong Balmer line emission spectrum during the *IUE* era, though its strength had been steadily decreasing from 1972 (Slettebak 1982). The observed profiles are a coaddition of SWP 7753 and SWP 15059. The large number of shell lines in the FUV made it difficult to achieve a good continuum normalization. C iii 1176 Å is somewhat weak in the core. C iii 1247 Å suggests a solar abundance. The N iii 1183-1184.5 Å multiplet should be well-defined in a star with a photospheric temperature of 27 kK but this spectral region contains a large number of weak shell lines, which suppress the flux and introduce problems with the continuum normalization. There is no convincing evidence that the photospheric nitrogen in this star has a non-solar value.

## The C/N abundance in massive Algol binaries

In Sect. 5 we have seen that carbon underabundance is commonplace, at least from profile fits to the C iii 1176 Å multiplet in seven of the eight Be stars that we chose for this investigation. On the other hand, we cannot confirm N-enhancement in these same stars.^9^ Relevant information may be extracted from Algol-type binaries since they might represent a pathway to Be star creation, and indeed evidence for CNO processing is observed in the Algol binary RY Per (B3Ve + F7 II-III, P = 6.86 d), which is probably undergoing Case B (or Case BB) mass transfer.

**Fig. 5:**
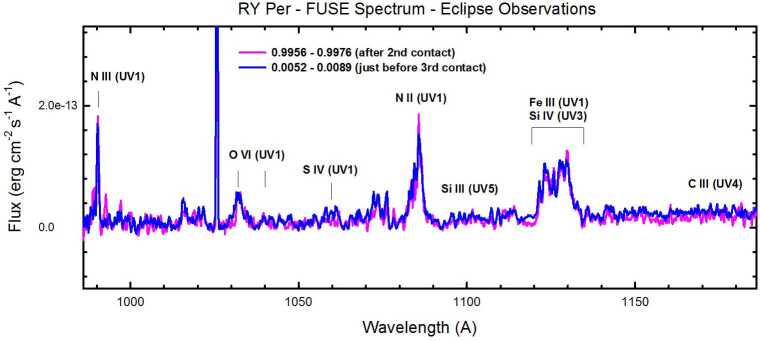
The *FUSE* spectrum of RY Per observed during total eclipse. Note the strong emission lines of nitrogen and the absence of C iii 1176 Å

The spectrum of RY Per observed with the *FUSE*^10^ spacecraft during the total phase of its eclipse is shown in Fig. 5. The only features observed are circumstellar emission lines from ionized species that range from O vi (formed in a 300 kK plasma) to N ii that is typically found in a plasma of moderate temperature, probably in the region of the gas stream impact in this case (Barai et al. 2004). Notably absent is emission from C iii 1176 Å. The N ii resonance multiplet at 1084 Å appears unusually strong, so this system may represent the abundance pattern of CNO processing, not requiring any additional depletion of N as was required in some Be stars.

**Fig. 6:**
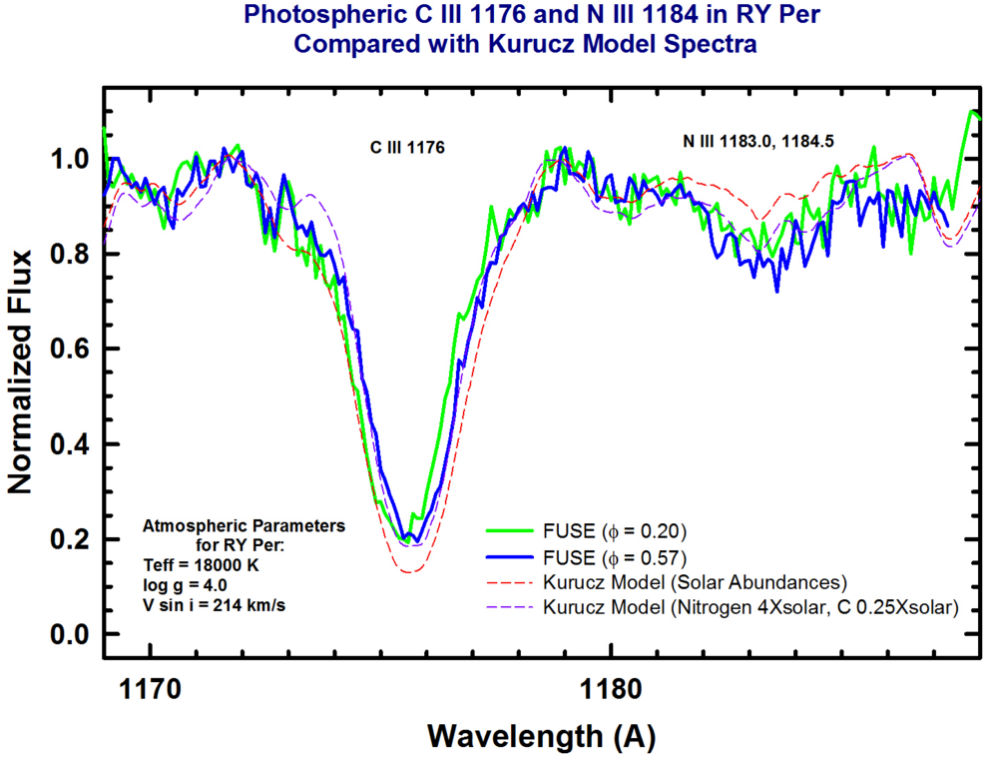
Comparison between the observed line profiles of the C iii 1176 Å and N iii 1184 Å multiplets in RY Per with the model calculations

Outside of eclipse an apparently normal, rotationally-broadened B3 spectrum is observed. There is no trace of the emission lines seen during totality (cf. *IUE* spectrum of the 1170-1185 Å region in Fig. 6). Although the rotational velocity of the B3 star is large (probably due to angular momentum gain during the mass transfer process), there is an obvious broad feature centered on the N iii 1183.0, 84.5 Å doublet. Comparison of the observed spectrum with model calculations reveals clear evidence for N enhancement. Furthermore, the C iii 1176 Å multiplet shows evidence of C depletion in the photosphere. The B3e primary in this system shows clear evidence of having gained material from the core of the transformed secondary.

Carbon deficiency in individual Algol primaries was reported as early as the 1980s. Parthasarathy et al. (1983) performed an abundance analysis of CH and CN lines in the secondaries of U Cep and U Sge and found a [C/Fe] value of −0.5, and a [N/C] enhancement of +0.5. Later Cugier and Hardorp (1988) analyzed the C ii resonance multiplet 1335 Å observed in $\beta $ Per and $\lambda $ Tau using *IUE* archival spectra and found underabundances of [C/H] = −3.87 and −3.88, respectively. Polidan (1988) found no evidence of carbon in ionization stages 1-4 in simultaneous *IUE* and *Voyager* observations of the massive binary V356 Sgr. The latter conclusion was confirmed by Tomkin and Lambert (1994). Neither of the aforementioned authors commented about nitrogen. Underabundances of C were also found in eight other Algol binaries from an analysis of the C ii 4267 Å line (Tomkin et al. 1993)^11^

It seems possible that C underabundance is the best diagnostic of previous CNO processing, since only the extremely high-$T$ triple alpha process can replenish the carbon once lost, and the products of such high-$T$ processing might be less apt to appear at the surface of the donor star. Enhancement of N is a less certain requirement for claiming CNO processing, because there are additional processes that could occur subsequently to reduce the N abundance, such as a stripped-star analog of “hot bottom burning” where convection could carry the envelope N into regions of exposure to hot alpha particles that are not necessarily hot enough to undergo triple alpha fusion of C. Since capture of alpha particles can convert N-14 to O-18 and Ne-22 at lower $T$ than triple alpha, the transferred gas observed in some Algol primaries and many Be stars could have undergone C depletion without showing the concomitant N enrichment. This would only occur if the donor star evolves through core He burning prior to the final mass transfer episode, i.e., if case BB mass transfer occurs.

## Nitrogen enrichment: single or binary star evolution?

The question of nitrogen abundances in Be+sdOB systems connects directly to the broader issue of internal mixing in (single-star) massive star evolution. The most widely invoked mechanism is rotational mixing (Maeder and Meynet 2000), but the VLT-FLAMES survey (Hunter et al. 2008) revealed a more complex picture: while N enrichment generally increases with $v\sin i$, two striking anomalies emerge. One is a population of slowly rotating, N-enhanced stars, often linked to magnetic braking. The other is a set of rapid rotators showing little or no N enrichment, and also little change to C. So those stars could be attributed to the absence of mixing, suggesting the mass transfer history we were attempting to test in our galactic Be sample. But instead we find cases where there is little N enrichment but there does appear to be C depletion, raising a new puzzle about what happened to that C.

Since there does remain an overall trend of N enhancement at higher rotational velocity, there can still be a role for rotational mixing in predominately single star evolution, but it is becoming likely that binary evolution needs to be accounted for in order to explain the entire Hunter diagram. The classical Be stars in the galaxy may add a new wrinkle to this landscape, whereby mixing from nonstandard nucleosynthesis regimes in evolved stripped stars may also play a necessary role in destroying the N that CNO-cycle processing has turned the C into, though careful theoretical modeling is needed before this speculation can be promoted. Nevertheless, it is significant that Be stars often fail to exhibit N enrichment, despite being among the most rapidly rotating stars in the galaxy. This presents theoretical challenges, especially when C appears to be depleted. Further observations are needed to clarify the scope of this challenge.

## Progress through future UV spectroscopy missions

In the preceding sections, we have described how the abundance patterns observed in classical Be stars, particularly enhancements or anomalies in C and N, are key tracers of evolution, internal mixing, and of possible mass transfer events. However, further progress is presently observationally limited. Interpreting abundance patterns is complicated by the challenge of extracting surface abundance signatures in the presence of the confounding effects of rotation, viewing geometry, microturbulence, and circumstellar contamination.

As we have described, existing *IUE* and *HST* observations have enabled substantial progress, in particular with the discovery using *HST* of widespread sdO binary companions (e.g., Wang et al. 2021). However, *HST* has provided high-resolution UV spectra for only a small number of Be stars, typically limited to bright or well-known systems. Systematic C and N abundance analyses are available for only a handful of Be stars, and often lack sufficient temporal sampling to properly understand the effects of rapid rotation and circumstellar emission contamination. *HST*’s high oversubscription rate makes it impractical to conduct a systematic survey of Be stars, particularly across different spectral types, inclination angles, and evolutionary states. The archival *IUE* sample, while broader, suffers from limited spectral resolution and SNR. A new, dedicated FUV facility is needed to attack Be star abundances and evolution.

### The need for FUV spectroscopy

A full test of how binary mass transfer might be affecting C, N, and Ne abundances requires the benefit of the many FUV lines of these elements, with support from ground-based optical determinations of the O abundances. One such concept for a future FUV spectroscopy mission with sufficient resolution and dedicated monitoring of Be stars is the *Polstar*, a NASA Small Explorer (SMEX) FUV spectropolarimetry mission concept (Drake et al. 2025; Scowen et al. 2025). If selected, this mission would launch in the early 2030s, enabling time-domain, high-resolution ($R = 20{,}000$) spectropolarimetry with the fidelity and cadence required for advancing our understanding many aspects of Be stars, including these abundances, using lines such as the strong N v 1238,1242 Å doublet, the C iv 1548,1550 Å doublet, the strong C iii 1176 Å multiplet, C iii 1247 Å, as well as the N iii 1184 Å and 1750 Å multiplets, as well as other transitions that are inaccessible or blended in the optical region. These lines arise in the upper photosphere or inner wind and are sensitive to the chemical enrichment of the stellar surface, including N enhancement from rotational mixing or mass accretion.

Compared to optical diagnostics, UV spectroscopy is less affected by disk continuum veiling and can provide abundance determinations even for rapid rotators where extreme line broadening obscures traditional photospheric features. The diagnostic value of UV CNO lines has been demonstrated in synthetic populations (Leitherer et al. 2014) and applied to interpreting chemical signatures in rapidly rotating stars of varying evolutionary histories. The key is a complete accounting of all the fusion endpoints including C, N, O, and Ne, to ascertain the conditions under which the fusion occurred to distinguish model outcomes. Also essential to making progress will be high signal-to-noise spectra of a statistically significant sample of Be stars at low and moderate inclination, expanded well beyond our archival sample to establish correlations.

### Polarization and photospheric vs. Disk contributions

A perennial challenge in abundance studies of Be stars is the contamination from circumstellar emission and scattering, especially in the UV where electron scattering in the disk can veil or distort line profiles. The ability to measure linear polarization would provide independent information to help overcome this limitation because linear polarization in the UV is more sensitive to the geometry of the disk than it is to the oblateness of the rotating photosphere. Polarization also depends on inclination, helping to interpret rotational $v\sin i$, enabling separation of the photospheric light from scattered contributions. Moreover, line polarization—including the so-called “line effect”, where polarization decreases across emission lines—provides a robust diagnostic of the spatial origin of specific spectral features (Ignace et al. 2025, 2022). Thus, polarization information can assist in mapping the geometry and optical depth of the circumstellar disk in support of line-strength measurements, allowing modelers to isolate the intrinsic stellar flux and perform more accurate abundance analyses.

### Polarization and binary interaction scenarios

Understanding whether observed C and N surface abundances in Be stars are affected by internal rotational mixing or past binary interaction is an important question in modern stellar evolution theory that requires higher resolution and/or better precision measurements of a wide array of rapidly rotating massive stars. Independent FUV polarization information can contribute stellar inclination and oblateness constraints, and thus quantify the degree of critical rotation (Scowen et al. 2022). These measurements help break the $v\sin i$–inclination degeneracy and enable precise determinations of equatorial rotation velocity and gravity darkening effects (Cotton et al. 2017).

Simultaneously, the UV spectroscopic capabilities of a dedicated massive-star mission could detect the narrow-lined signature of stripped subdwarf (sdO) companions in Be+sdO binaries (Jones et al. 2022), even when these contribute only ∼0.1–0.5% of the UV flux. The combination of companion detection and stellar rotation measurements allows a systematic comparison of abundance anomalies with rotation and binarity—precisely what is needed to establish when C and N abundances are controlled by single-star evolution or binary mass transfer, in both Be and Bn stars (Jones et al. 2022; Scowen et al. 2025).

## Concluding comments

This paper is a preliminary archival and purely spectroscopic investigation of the issues that a broader future investigation will likely encounter. The C iii 1176 Å feature observed in seven of the eight program stars analyzed here suggests an underabundance of photospheric carbon. This result is compatible with those of several other studies of the chemical composition of Be star atmospheres since the late 1970s, e.g., Peters (1979), Dunstall et al. (2011), Kolbas et al. (2014), Ahmed and Sigut (2017), Dufton et al. (2024), and it also is consistent with several abundance studies of B-type Algol primaries (mostly in the *IUE* era, cf. Sect. 6). This result suggests evidence of CNO processing, consistent with the idea that many Be stars could be spun up by mass transfer from an evolved and stripped companion.

Along with that relatively normal finding comes the expectation that CNO processing should also enhance N, but in general, only modest N enhancements have been reported for both Be stars and the early B-type Algols. This presents a puzzle given how common are C underabundances in these stars. Of course, one would not expect to observe a pure CNO-processed photosphere because the material from the Be star’s core and also the transferred material would mix with some non-processed photospheric C and N. Nevertheless, when C is missing, we normally expect it to be converted almost completely into N, but an emerging finding that is underscored by our study is that in many cases associated with possible mass transfer, the number of C + N atoms appears to drop. As such, CNO processing does not appear to be the final chapter in the explanation of the abundances. This presents an acute observing challenge to support or refute this result, and if supported, an acute theory challenge to establish the cause.

Current expectations surrounding CNO abundances and mass transfer suggest that the degree of abundance effects should depend on the epoch of mass transfer being considered. Early in the post mass-reversal evolution, the delivery of the gas stream material is by direct impact, in which the stream burrows deep into the photosphere. During the late period of mass transfer, the deeper layers of the mass loser are being peeled off, so one would expect to see larger N abundances if the gas is being pulled from CNO processed zones. These enhancements would come from conversion of C to N in the CNO I cycle, and possibly also O to N in the CNO II cycle, so one expects the gainer to show a N increase with age in the transfer process. Since this is apparently not what we observe, it suggests an additional process may appear, perhaps in the final stages of mass transfer.

The abundance anomaly we report is tentative due to observational uncertainties and a small set of examples, but if it is upheld in future broader high-precision surveys, and if its explanation lies in the binary mass transfer history, then it could be due to nonstandard nucleosynthesis products being transferred from the stripped companion in the late stages of mass transfer. Since the answer appears to involve N depletion, we speculate that the answer might lie in the hot alpha-particle layers outside the carbon core in late evolutionary stages of the mass donor, that can convert N-14 to O-18 and possibly Ne-22.

In particular, if the stripped companion’s core reaches a mass $\mathrm{> 0.5}$ solar (which should be common for Be stars given their high mass), then the He will eventually burn into a C/O core with a hot triple-alpha shell similar to AGB stars, albeit without the core degeneracy and with a much weaker H envelope. Nevertheless, if such an evolved stripped star retains enough of a hydrogen envelope, it can expand for similar reasons as AGB stars, crossing its Roche lobe for the second time and initiating case BB transfer. Then the evolved stripped star could experience deep envelope convection and mix to the surface N depleted material originating from hot $(>100 MK)$ regions that were not dense enough for significant triple-alpha creation of C, from the trail of ashes left by the receding triple-alpha burning core of this stripped AGB star. That zone of N depletion is the place we look for our possible answer as to how the sum of C + N can drop.

If this is occurring, the process would also exhibit enhancements in O-18 and Ne-22, though the first would be difficult to disentangle from the copious O-16. In contrast, the Ne-22 would be more easily noticed against the small Ne-20 abundance, but models would be needed to determine if enough Ne-22 could be generated to be seen against that background. Thus depletion in C + N remains the simplest signature of this potential process, and if supported, it would suggest that mass transfer in Algol-type binaries can continue through a kind of “AGB Algol” stage of case BB transfer, without requiring short periods.

Our overall picture requires either that semiconvection outside the carbon core in evolved stripped stars is not efficient enough to mix lower-density hot zones where the N is being converted to O into the high-density triple alpha fusion zone that would lead to C enhancements, or it would require that the N depletion occurs due to He thermal pulses farther out in the envelope. It also requires that case BB mass transfer from a carbon-core stripped donor can occur at wide separation, so the donor must retain an H-containing envelope long enough to combine large radius with deep convection of N-depleted material. That implies a limit on how strong the wind mass-loss rate from the stripped star can be. With these assumptions in place, then as case BB mass transfer proceeds at wide separations, this C + N depleted layer could represent the final stages of mass transfer, leaving behind the C-enriched core to become a white dwarf. This late stage mass transfer might also represent a significant final injection of angular momentum to help form a subsequent Be star.

The processes detailed above might also be relevant for studies of the formation of Be/X-ray binaries. Recently, there has been theoretical work on the formation of Be/X-ray systems that suggest low-kick supernovae result from ultra-stripped helium stars, and other supporting evidence for the possibility of case BB mass transfer (Richardson et al. 2023; Valli et al. 2025; Vinciguerra et al. 2020).

Detailed binary mass transfer simulations would be needed to establish the plausibility of the scenario presented in this section, but if verified as a potential outcome, it would allow abundance analyses of the surfaces of the mass gainer to be used to understand the evolution of the stripped star and the binary as a whole. It would also give insight into the origin of the rapid rotation of the high-mass Be star, or lower-mass Bn star, that is created by the binary mass transfer scenario. The mass of the gainer will relate to the mass of the stripped companion, which could have a significant impact on how these abundance anomalies are produced, and the fact that this cause of abundance anomaly requires mass transfer has implications for the rotation of the primary star. Whatever the explanation for these types of abundance anomalies, resolving the puzzle will provide important new constraints on the physics of stripped stars, whose nature has yet to be fully probed because of the difficulties in directly observing them.

## Data Availability

Data can be provided upon reasonable request to the corresponding author.

## Footnotes

1 In this paper, *massive* means that the primary is an early B star. Most of the sdO companions confirmed so far are in the 0.5-1.5 $\mathrm{M_{ \odot}}$ range.

2 The interior flows could be from meridional circulation due to rotation or other flows that have resulted from a direct impact of a gas stream in binary mass transfer. The direct impact scenario prevails during pre-mass-reversal and for about 40% of the system’s post-mass-reversal evolution (Peters et al. 2022).

3 Mikulski Archive for Space Telescopes, https://archive.stsci.edu.

4 Nitrogen abundances for many early Be stars have been determined from the analysis of the moderately-strong optical line N ii 3995 Å. These continue to be a good complement to our FUV studies and serve as an independent check of our results.

5 https://archive.stsci.edu/iue/manual/dacguide/.

6 Except for 16 Peg, which probably was diskless at the time of the *IUE* observations. If the Be star does not have an sdO companion, the inferred abundance will be too small.

7 From Grevesse and Sauval (1998).

8 See the *Copernicus* spectral atlases for $\tau $ Sco (Rogerson and Upson 1977), $\gamma $ Peg (Rogerson 1985), and $\iota $ Her (Upson and Rogerson 1980).

9 The same effect was reported for the *pole-on* Be star $\upsilon $ Cyg in Peters (1979).

10 The *Far Ultraviolet Spectroscopic Explorer* launched by NASA in 1999. An advantage of studying Algol binaries in the FUV is that the light from the late-type secondary does not significantly contribute to the system’s flux.

11 $[\mathrm{C}/\mathrm{H}]_{\mathrm{av}} = -0.32 \pm 0.15$ dex.
